# The Highly Efficient Expression System of Recombinant Human Prolidase and the Effect of N-Terminal His-Tag on the Enzyme Activity

**DOI:** 10.3390/cells11203284

**Published:** 2022-10-19

**Authors:** Justyna Czyrko-Horczak, Magdalena Nizioł, Antonella Forlino, Roberta Besio, Wojciech Miltyk

**Affiliations:** 1 Department of Structural Chemistry, Faculty of Chemistry, University of Bialystok, 15-245 Bialystok, Poland; 2 Department of Analysis and Bioanalysis of Medicines, Medical University of Bialystok, 15-089 Bialystok, Poland; 3 Biochemistry Unit, Department of Molecular Medicine, University of Pavia, 27100 Pavia, Italy

**Keywords:** human recombinant prolidase, protein expression system, prolidase activity

## Abstract

Prolidase is an enzyme hydrolyzing dipeptides containing proline or hydroxyprolineat the C-terminus and plays an important role in collagen turnover. Human prolidase is active as a dimer with the C-terminal domain containing two Mn^2+^ ions in its active site. The study aimed to develop a highly efficient expression system of recombinant human prolidase (rhPEPD) and to evaluate the effect of the N-terminal His-Tag on its enzymatic and biological activity. An optimized bacterial expression system and an optimized purification procedure for rhPEPD included the two-step rhPEPD purification procedure based on (i) affinity chromatography on an Ni^2+^ ion-bound chromatography column and (ii) gel filtration with the possibility of tag removal by selective digestion with protease Xa. As the study showed, a high concentration of IPTGand high temperature of induction led to a fast stimulation of gene expression, which as a result forced the host into an intensive and fast production of rhPEPD. The results demonstrated that a slow induction of gene expression (low concentration of inducing factor, temperature, and longer induction time) led to efficient protein production in the soluble fraction. Moreover, the study proved that the presence of His-Tag changed neither the expression pattern of EGFR-downstream signaling proteins nor the prolidase catalytic activity.

## 1. Introduction

Prolidase or peptidase D (PEPD) is an enzyme hydrolyzing dipeptides containing proline or hydroxyproline at the C-terminus [1]. It is ubiquitously distributed (e.g., erythrocytes, kidneys, fibroblasts [2], and platelets [3,4]) and plays an important role in collagen turnover. For many years, prolidase was considered an enzyme that could regulate intracellular processes via catalytic reactions. Perhaps this limitation in understanding its role was due to the loss or reduction of enzymatic activity resulting in PEPD deficiency (PD). PD is accompanied by hard-to-heal ulcers, splenomegaly, immune deficiency, and mental retardation [2]. To date, there is no successful therapy for PD and its pathogenesis remains unclear. However, recently discovered novel functions of PEPD as a ligand of EGFR and HER2 [5,6], the regulator of p53 activation [7], and the maturation of interferon α/β receptor IFNAR1 [8] have been reported. Further research into the role of prolidase as an intracellular signaling pathways modulator could increase the knowledge of the pathophysiology of PD and help to discover effective treatment.

Human prolidase is active as a dimer. Each of the two subunits is folded into two domains, with the C-terminal one containing the active site with the metal-binding site [9]. Human PEPD is catalytically and biologically active only when each of the two subunits contains two Mn^2+^ ions in its metal active site [10,11].

Conducting biological, biochemical, or structural research aimed at understanding the biological function of prolidase, explaining mechanisms of the activity occurring in living organisms, or designing therapeutic strategies requires a large amount of pure protein. For these purposes, effective systems are needed for the high-throughput production of the active protein of good stability and purity on a large scale. Formerly, prolidase was obtained from the tissues of living organisms, however, these methods were not effective. The tremendous progress of genetic engineering and the development of powerful molecular techniques has made it possible to develop and apply efficient systems for the production of recombinant proteins in vitro in the laboratory [12]. Their use allows for the efficient production of these peptides, which in natural conditions are present in insufficient amounts. Various protein expression systems are used for the production and purification of recombinant proteins. These include bacterial, yeast, insect, and mammalian expression systems. So far, human recombinant prolidase has been produced mainly in expression systems based on eukaryotic or bacterial cells. Bacterial cultures have been used more often because, as stated, they are not as demanding as the eukaryotic cell culture system and provide better performance in terms of production [13]. Recombinant human prolidase was expressed not only in (i) bacterial cells, e.g., in *E. coli* [6,9,13], but also in *Pichia pastoris* [14], *Saccharomyces cerevisiae* [15], and (ii) *Trichoplusiani* larvae [8]. There are also known methods of isolating prolidase from isolated mammalian tissues [16,17,18,19,20] and bacterial cells, e.g., *Lactobacillus* [21].

The purpose of the present study has been to develop a highly efficient expression system of recombinant human prolidase and to evaluate the effect of the N-terminal His-Tag on its enzymatic and biological activity.

## 2. Materials and Methods

### 2.1. Expressionof rhPEPD (Optimized)

The formulation of rhPEPD kindly provided by Prof. Antonella Forlinowas used to transform *E. coli* BL21(DE3) competent cells (Invitrogen, Waltham, MA, USA) using the heat shock method [4]. For this purpose, 1 µL of the formulation (25 ng/µL) was added to 100 µL of competent cells and mixed gently with the tip. The mixture was cooled for 5 min on ice, heated for 60 s at 42 °C, and cooled back for 5 min on ice. A quantity of 1 mL of LB (Bioshop, Burlington, ON, Canada) was added to the transformed cells and incubated for 60 min at 37 °C with gentle mixing. These transformed cells were used to prepare the overnight culture. The transformed cells were added to 15 mL of LB medium (Bioshop, Burlington, ON, Canada) with the addition of 100 μg·mL^−1^ ampicillin (Bioshop, Burlington, ON, Canada) and grown at 37 °C with shaking to 200 rpm for 13 h. The overnight culture was used for the inoculation of 1 L of LB broth (Bioshop, Burlington, ON, Canada) with ampicillin (100 μg·mL^−1^) (Bioshop, Burlington, ON, Canada). The prolidaseexpression in *E. coli* cells was inducted by the addition of Isopropyl-d-thiogalactopyranoside (IPTG) (Bioshop, Burlington, ON, Canada) attwo different concentrations of 1 mM and 0.2 mM. Induction with a high IPTG concentration (1 mM) was carried out at different times and temperatures (37 °C for 2 h, 30 °C for 5 h, 25 °C for 10 h, 18 °C for 15 h, and 16 °C for 15 h), whereas induction with a low IPTG concentration (0.2 mM) was performed at 30 °C for 5 h, 25 °C for 10 h, and 18 °C for 18 h. After induction, the culture was cooled to 4 °C for 1 h with gentle mixing (100 rpm) followed bycentrifugation (15 min, 4500 rpm, 4 °C). Then, the cell pellet was rinsed twice with a lysis buffer containing 300 mM NaCl, 20 mM Tris-HCl pH 8.0, 20 mM imidazole, 1 mM EDTA, and 10% glycerol. The cell pellet was resuspended in a lysis buffer containing the appropriate components described as follows: Pellets from induction with 1 mM IPTG were suspended in a lysis buffer with the addition of lysozyme (100 µg µmL^−^^1^) (Bioshop, Burlington, ON, Canada). The lysate was left for 1 h at 4 °C and gently mixed (100 rpm) to dissolve the pellet. Then, two factors releasing protein from the inclusion bodies were added into the solution: N-laurylsarcosine at a final concentration of 1.5%, and Triton X-100 at afinal concentration of 2% (conditions with 1 mM IPTG). This solution was incubated for 1 h at room temperature. Pellets after induction with 0.2 mM IPTG were dissolved in the lysis buffer with the addition of lysozyme (100 µg/mL) and Tris(2-carboxyethyl) phosphinehydrochloride (TCEP) (1 mM) and this solution was left for 1 h at 4 °C with gentle mixing (100 rpm). Then, all the collectedlysates were frozen at −80 °C. After quick defrosting, the lysates were sonicated on ice for 4 min (pulse of 3 s, the interval 20 s) and centrifuged for 30 min at 15,000 rpm at 4 °C. The supernatant and precipitate were analyzed by SDS–PAGE applying a 10% polyacrylamide gel.

### 2.2. Purification, Activation, and His-Tag Removal of rhPEPD

The supernatant was loaded onto a HisTrap column (Bio-Rad, Hercules, CA, USA) with Ni-NTA affinity resin (IMAC) equilibrated with 0.1 M NiSO_4_ for the purification of polyhistidine-tagged proteins. Then, bound protein was washed three times with a lysis buffer and then elutedwith an elution buffer containing 300 mM imidazole, 300 mM NaCl, 20 mM Tris-HCl, pH 8.0, 20 mM imidazole, 1 mM EDTA, 10% glycerol, and 1 mM TCEP. The eluent was dialyzed against the lysis buffer for 12 h at 4 °C. The eluted protein was analyzed by SDS–PAGE, using a 10% polyacrylamide gel. To improve the purificationof the preparation, we decided to add a second purification step tothe process. The eluted mixture was concentrated to 10 mL using ultracentrifugation filters Amicon-Ultra10 (Merck Millipore, Burlington, MA, USA) and loaded onto a Superdex 200 (Pharmacia, Jersey, NJ, USA) gel filtration column equilibrated with an FPLC buffer (150 mM NaCl, 25 mM Tris-HCl pH 7.5, 1 mM TCEP-HCl). The protein as a dimer was eluted with a buffer of FPLC in one peak corresponding to a molecular mass of 114 kDa. A commercially available standard was used to determine the protein mass (Bio-Rad, Hercules, CA, USA). Fractions with rhPEPD were concentrated to 1–1.5 mg/mL, using Amicon Ultra 10 filters, and subsequently passed through a 0.22 μm filtration membrane. The obtained inactive rhPEPD preparation was activated by incubation with 1 mM Mn^2+^ ions at 37 °C for 1 h. The Mn^2+^ ions were removed by dialysis against an FPLC or a PBS buffer for 12 h at 4 °C and next passed through a 0.22 μm filtration membrane.

Factor Xa was used to cleave the His-Tag, and two cleavage procedures were used. The first one was during protein affinity purification on the nickel column, and the second one was after the FPLC purification of the protein. In both steps, Factor Xa was removed from the solution by affinity capture on Xarrest™ Agarose (Merck Millipore, Burlington, MA, USA) according to the Xarrest manufacturer indication. All buffersused for purification were pH adjusted at 4 °C, filtered on 0.22 µm filters, and stored at 4 °C in shade.

### 2.3. Protein Electrophoresis (SDS-PAGE)

To demonstrate the purity of recombinant rhPEPD, 10% SDS-PAGE was used, according to Laemmli’s method [22]. The samples were prepared in the loading buffer (with glycerol, SDS, and 0.01% *w*/*v* bromophenol blue) and then heated for 5 min at 95 °C. The protein was loaded on the gel at a concentration of 0.1–1 mg/mL. A commercial standard (Bio-Rad, Hercules, CA, USA) was used to monitor the protein separation and its mass.

### 2.4. Dynamic Light Scattering (DLS)

Dynamic light scattering (DLS) experiments were carried out to evaluate the purity of the protein preparation. Before measuring the protein solution, the sample was filtered by 0.22 µm and stored at 4 °C. The measurement was performed at 22 °C. The concentration of protein was 1 mg/mL and three independent repetitions of the analysis were performed.

### 2.5. Mass Spectrometry

Mass spectrometry measurements were carried out with SYNAPT MS (Waters, Manchester, UK) coupled to an Acquity UPLC liquid chromatograph (Waters, Manchester, UK) in the Environmental Mass Spectrometry Laboratory, Institute of Biochemistry and Biophysics of the Polish Academy of Sciences (Warsaw, Poland). Mobile phase A was water; mobile phase B was acetonitrile. The flow was 0.1 µL/min, the gradient profile was 0 to 2 min, linear gradient elution 0% B; from 8 min 40% B, from 9 to 11 min 90% B, from 12 min 1% B. The capillary voltage was 2.5 kV; the source was kept at 80 °C; desolvation temperature was 150 °C; cone gas flow, 150 L/h; and desolvation gas flow, 650 L/h.

### 2.6. HaCaT Cell Cultures

HaCaT cells were chosen as a cellular model and were supplied by Cell Line Service (Eppelheim, Germany). Cells were cultured in DMEM (PanBiotech, Aidenbach, Germany) supplemented with 10% fetal bovine serum (FBS; Gibco, Carlsbad, CA, USA) and 1% penicillin/streptomycin (Gibco, Carlsbad, CA, USA) at 37 °C and of 5% CO_2_.

### 2.7. HaCaT Treatment

The cells (3rd–5th passages) were incubated with His-Tag and non-His-Tag recombinant human prolidase at the concentration of 10, 25, and 50 nM. Cells were cultured in a DMEM cell culture medium without FBS for 30 min and 24 h. For Western immunoblotting and prolidase activity, cells were cultured on 100 mm dishes at a density of 2 × 10^6^ cells/plate.

### 2.8. Preparation of Lysates

Once cells were washed with cold PBS (pH 7.4), then a RIPA lysis buffer (Thermo Fisher Scientific, Waltham, MA, USA) containing protease inhibitor (cOmplete™ Protease Inhibitor Cocktail, Roche, Basel, Switzerland), phosphatase inhibitor cocktail (PhosSTOP, Roche, Basel, Switzerland), and viscolase (A&A Biotechnology, Gdańsk, Poland) were added. After 15 min incubation on ice, the fresh supernatant was collected and stored at −80 °C. The Pierce BCA assay kit (Thermo Fisher Scientific, Waltham, MA, USA) was used for protein concentration.

### 2.9. Western Immunoblotting

A quantity of 20 µg of protein diluted ina lysis buffer and mixed with a Laemmli buffer containing 5% β-mercaptoethanol (Sigma Aldrich, Saint Louis, MO, USA) was denatured at 95 °C for 7 min. Then, 10% SDS-PAGE gels were loaded and the proteins were blotted onto PVDF membranes (BioRad, Hercules, CA, USA). The membranes were incubated with 5% non-fat dried milk (Santa Cruz Biotechnology, Dallas, TX, USA) or BSA (Sigma Aldrich, Saint Louis, MO, USA) in TBS-T (20 mM Tris-HCl, 150 mM NaCl, 0.1% Tween-20, pH 7.6) on an agitator for 1 h at RT. Then, the membranes were agitated with primary antibodies (Table 1) overnight at 4 °C and subjected to alkaline phosphatase-linked goat anti-rabbit antibody (1:10,000, Sigma-Aldrich, Saint Louis, MO, USA). After washing with TBS-T for 5 min thrice, the protein bands were visualized with 1-Step™ NBT/BCIP Substrate Solution (Thermo Fisher Scientific, Waltham, MA, USA). The intensities of protein bands were semi-quantitatively calculated with ImageJ software (ver. 1.53s, https://imagej.nih.gov/ij/; accessed on 21 June 2022). All protein analyses were run in triplicates.

### 2.10. Determination of ProlidaseActivity

The activity of prolidase was determined according to the method published by Besio et al. [23]. TECAN Infinite^®^ M200 PRO (Männedorf, Switzerland) was used for the measurement of absorbance at 515 nm.

### 2.11. Statistical Analysis

All experiments were carried out at least in triplicates. Data are shown as a mean ± SD. For statistical analysis, a *t*-test or one-way analysis of variance (ANOVA) with Bonferroni’s correction was used and a *p*-value < 0.05 was considered significant. Statistical significances were expressed using symbols such as *, ^#^ < 0.05; **, ^##^ < 0.01; ***, ^###^ < 0.001; and ****, ^####^ < 0.0001 (* vs. His-Tag rhPEPD control, ^#^ vs. rhPEPD control). Statistical analysis was performed using GraphPad Prism 5.01 (GraphPad Software, San Diego, CA, USA).

## 3. Results

### 3.1. Enzyme Expressionand Purification

The previously described procedure to obtain rhPEPD in bacteria allowed only limited recovery of the protein in the soluble fraction. The protein was mainly present in inclusion bodies, i.e., aggregates of non-native conformation. The use of several refolding techniques for recovering biologically active protein from inclusion bodies was not effective. We used, among others, the addition of N-laurylsarcosine (1%, 1.5%) and Triton X-100 (2%), but the protein release efficiency from the inclusion bodies was very low since we were losing most of the protein during refolding. To optimize the expression levels of rhPEPD, several conditions were tested (Table 2).

**Figure 1:**
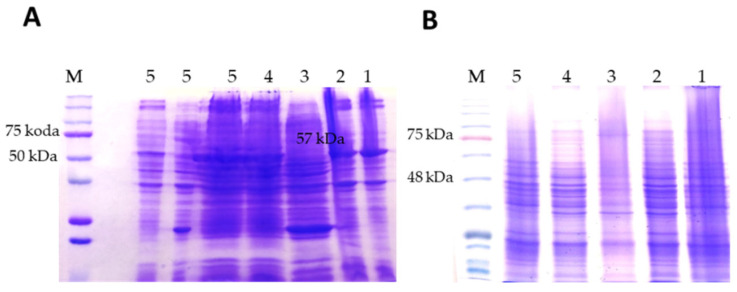
Gel electrophoresis analysis of the expression of rhPEPD. Dominant bands were identified by the mass size corresponding to the 57 kDa of rhPEPD as a monomer. Expression with 1 mM IPTG: lines 1–5 correspond to attempts 1–5; (**A**) the pellet after sonication of bacterial culture was analyzed (protein in inclusion bodies); (**B**) soluble fraction.

Different temperatures and induction times and two IPTG concentrations (Figure 1) were tested. In detail, our attempts focused on the extension of induction times while reducing both the temperature and the inducer (IPTG).

The protein was expressed but produced in inclusion bodies when 1 mM IPTG was used at various temperatures and induction times. SDS-PAGE analysis showed no protein in the soluble fraction (Figure 1B) with the presence of protein in the pellet after the centrifugation of the sonicate (Figure 1A).

**Figure 2:**
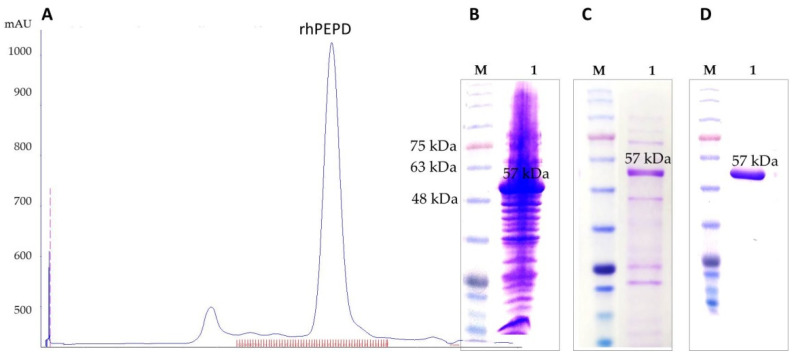
Results of purification and initial characterization of rhPEPD. (**A**) Chromatogram of HiLoad Superdex 200, the final size-exclusion chromatographic purification step recorded at 280 nm. The major peak corresponds to the monomeric form of rhPEPD with an approximate molecular mass of ≈50 kDa. SDS-PAGE analysis of rhPEPD. M—molecular weight marker: (**B**) rhPEPD after sonication to soluble fraction before Ni-NTA IMAC step; (**C**) rhPEPD after Ni-NTA IMAC step; (**D**) rhPEPD after size-exclusion chromatographic separation to the major peak of chromatographic fractions.

Figure 2A shows a chromatogram of HiLoad Superdex 200 in which the major peak corresponds to the monomeric form of rhPEPD with an approximate molecular mass of ≈50 kDa. Lowering the temperature and the induction factor, as well as extending the induction time, allowed for the production of protein in the soluble fraction (Figure 2B). Indeed, we obtained the highly soluble expression efficiency of the protein at 18 °C for 18 h with 0.2 mM IPTG concentration.

The soluble rhPEPD was purified by IMAC. We started our sonicates purification using 150 mM NaCl, but during the first purification step followed by dialysis, we noticed that the protein was partially precipitated. Therefore, we increased the salt concentration to 300 mM and maintained it until the gel filtration step. This maintains in solution the recombinant proteinon the first step of purification during Ni-NTA IMAC (Figure 2C). As a result, a high-purity protein preparation was obtained, but impurities in the form of other proteins were still present, requiring the optimization of an additional gel filtration purification step (Figure 2D).

As a consequence, we obtained a protein preparation of very high purity (Figure 2D), but with reduced catalytic activity (the enzyme requires the presence of a cofactor, Mn^2+^ ions). Therefore, the next step was incubation with a cofactor, which allowed for obtaining a fully active protein. During all steps of the purification procedure, we used buffers with added glycerol [24,25] to increase the stability of proteins and TCEP in order to reduce protein loss. The optimization of expression and purification conditions allowed for obtaining large amounts of protein in the soluble fraction of approximately 45–50 mg from 1 L of bacterial culture.

### 3.2. His-Tag Removal

We tested two different His-Tag removal methods for a purified protein solution. For this, we used protein with an initial concentration of 1.5 mg/mL. First, we directly added the Xa factor to the solution, which was then removed by Xarrest agarose. A loss of approximately 50% of the protein was observed using this procedure (we obtained a preparation with a concentration of approximately 0.8 mg/mL). Therefore, the His-Tag removal was attempted during the first stage of purification on a nickel column. For this purpose, two identical portions of sonicate were used. The first portion was purified without cutting the His-Tag on the nickel column. We used the second portion for His-Tag removal on the nickel column and carried out purification with cleavage in this step. The yield was much higher and the loss of protein was minimal (we obtained a preparation with a concentration of approximately 1.5 mg/mL).

### 3.3. Mass Spectrometry and SDS-PAGE

**Figure 3:**
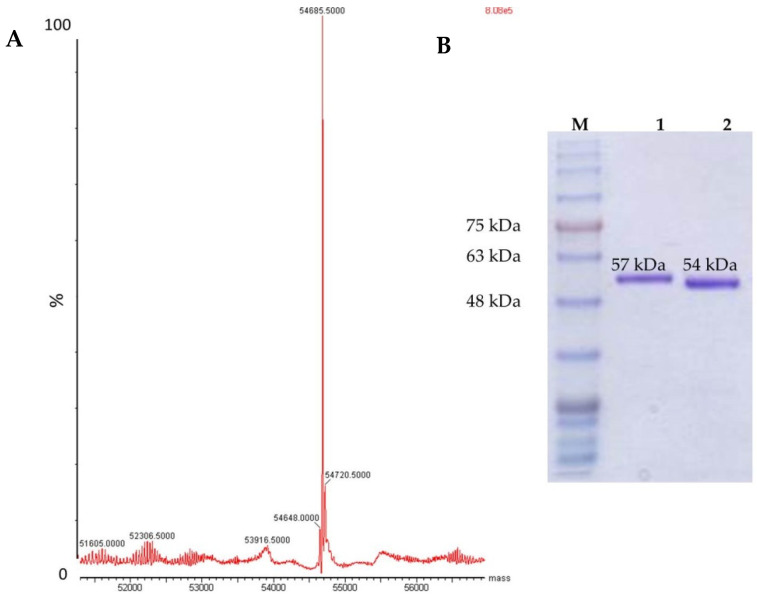
(**A**) Mass spectrometry of rhPEPD after His-Tag removal. (**B**) SDS-PAGE analysis, M—molecular mass markers, lane 1—rhPEPD with His-Tag, and lane 2—rhPEPD without His-Tag (after its removal with Xa protease).

Based on the rhPEPD sequence, the molecular mass of the protein with His-Tag (57.07 kDa) and protein without His-Tag (after cleavage with protease Xa) (54.68 kDa) was calculated. A high-resolution mass spectrometry technique (SYNAPT MS) was used for the analysis of protein mass (Figure 3). The mass spectrum of the rhPEPD contains one major peak, corresponding to the calculated molecular mass predicted from the amino acid sequence as 54,685.5 Da (Figure 3A). SDS-PAGE analysis indicates rhPEPD with the His-Tag band as ~57 kDa band, protein without His-Tag (after cleavage with protease Xa) band as ~54 kDa band (Figure 3B).

### 3.4. DLS—Confirmation of the Stability and Quality of the Purified rhPEPD Preparation

To confirm the quality of the protein, detect aggregates and determine the size of macromolecules in the protein solution, Dynamic light scattering (DLS) was applied.

**Figure 4:**
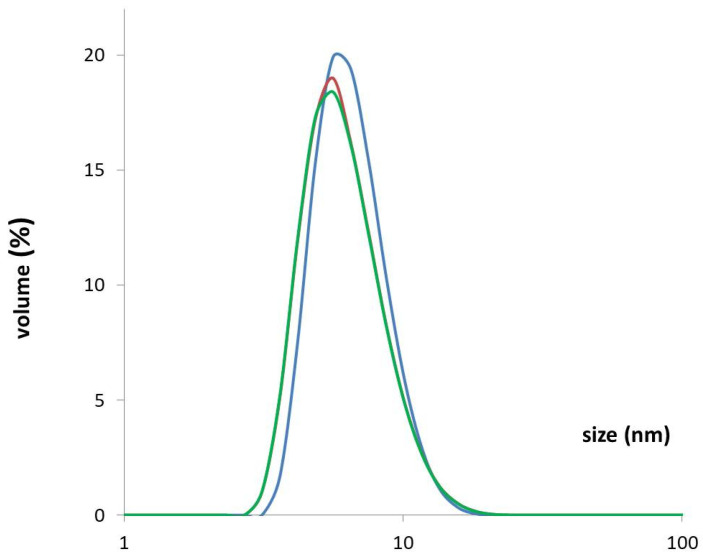
DLS measurement results for rhPEPD as the averages of three independent experiments for each protein sample. Measurement immediately after the two-stage protein purification (blue line), after 4 h of storage at room temperature (red line), and after freezing and thawing the preparation (green line).

The DLS method is extremely sensitive to the presence of aggregates and shows the quality of the preparation (Figure 4). The measurement of DLS for rhPEPD after freezing and thawing the protein in PBS solution showed no differences from the non-frozen protein preparation in an FPLC buffer tested right after purification. The measurement results indicated that the protein solution was monodisperse suggesting the absence of aggregate formation and that the quality of the protein preparation was good. The protein after purification could be frozen at −80 °C. Recombinant protein monodisperse size distribution was estimated from DLS as a hydrodynamic radius of 6.72 ± 0.59 nm.

### 3.5. Effect on the Enzymatic Activity of the N-Terminal-Fused His-Tag

**Figure 5:**
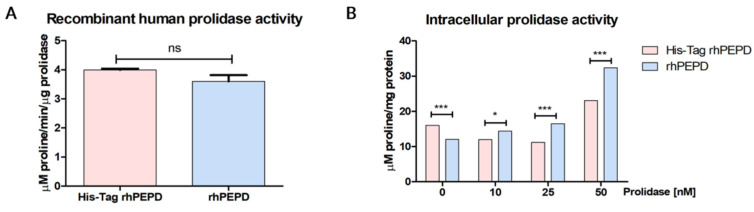
(**A**) The comparison of catalytic capabilities of His-Tag rhPEPD and rhPEPD. (**B**) The effects of extracellular His-Tag rhPEPD and rhPEPD on intracellular prolidase activity in HaCaT cells. Statistical significances were expressed using asterisks such as * < 0.05, and *** < 0.001; ns—not significant.

Prolidase activity was assessed by a determination of the release of proline from Gly-Pro by the recombinant enzyme according to Besio’s method [23]. As can be seen in Figure 5A, in terms of released proline from Gly-Pro, both His-Tag rhPEPD and rhPEPD were equally efficient. We next investigated whether extracellular prolidase affects intracellular enzymatic activity. We incubated human keratinocytes (HaCaT cells) with 10–50 nM prolidase. The results showed that extracellular prolidase stimulated its intracellular enzymatic activity. However, the effects were more potent in rhPEPD-treated HaCaT cells compared with His-Tag-rhPEPD treated keratinocytes. Nevertheless, both forms were able to stimulate intracellular activity showing a similar tendency (Figure 5B).

### 3.6. Effect on the Biological Activity of the N-Terminal-Fused His-Tag

**Figure 6:**
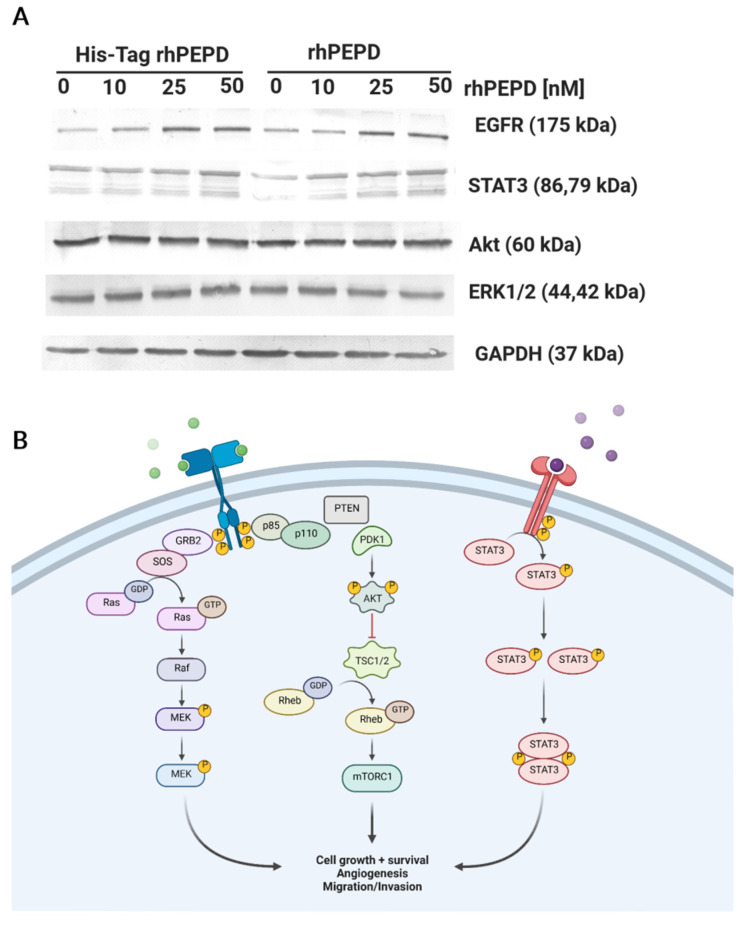
(**A**) The presence of His-Tag in the rhPEPD molecule does not affect the pattern of the protein expression downstream to EGFR. HaCaT cells were treated with His-Tag rhPEPD or rhPEPD for 24 h. (**B**) Prolidase-dependent EGFR-downstream signaling pathways. Created with Biorender.com (accessed on 21 June 2022).

It is well known that prolidase may act as a ligand of the epidermal growth factor receptor (EGFR) [6] and induce intracellular signaling promoting anabolic processes. Since prolidase stimulates EGFR, we tested whether and how the presence of His-Tag impacts the selected EGFR-downstream signaling pathways. To investigate its possible effects on the aforementioned proteins, we performed Western immunoblotting. The data demonstrated that after 24 h incubation with prolidase, the level of EGFR expression was significantly increased in a dose-dependent manner. The stimulating signal was transmitted to the downstream proteins such as Akt, STAT3, and ERK1/2. However, the levels of expression of these proteins did not depend on prolidase concentration. Additionally, the pattern of expression of all tested proteins was not changed between treatment with His-Tag rhPEPD and rhPEPD (Figure 6A). Figure 6B presents prolidase-mediated EGFR-downstream signal transduction.

**Figure 7:**
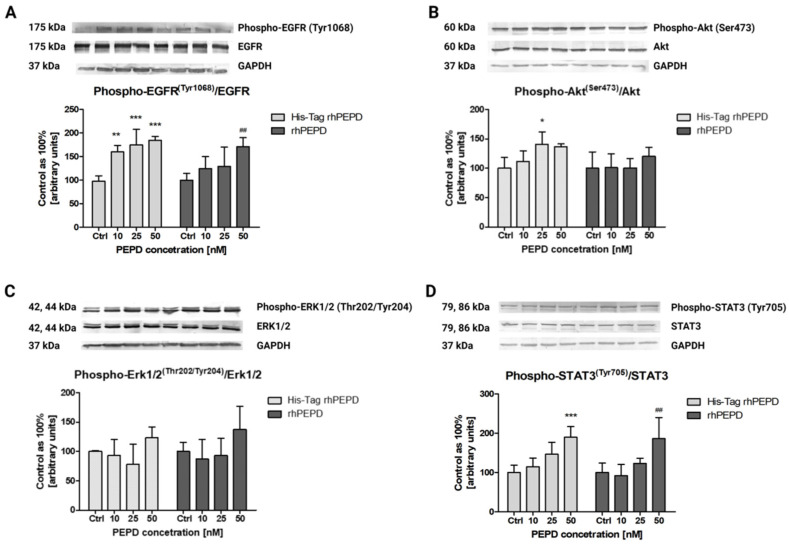
Phosphorylation of the selected EGFR-downstream signaling proteins such as (**A**) EGFR; (**B**) Akt; (**C**) ERK1/2; (**D**) STAT3 upon 30 min treatment with His-TagrhPEPD or rhPEPD. The ratio presents the level of phospho-protein to total protein expression. Statistical significances were expressed using symbols such as * < 0.05; ** < 0.01; *** < 0.001; ^##^ < 0.01 (* vs. His-Tag rhPEPD control).

The phosphorylation of EGFR at Tyr1068 occurred after treatment with both His-Tag and non-His-Tag rhPEPD. The ratio of phospho-protein to total protein showed that EGFR was activated strongly by His-Tag rhPEPD (Figure 7A). To observe EGFR-downstream signaling upon stimulation with prolidase (His-Tag and non-His-Tag rhPEPD), we chose three pathways: Akt, ERK1/2, and STAT3. The differences in total protein expression levels between His-Tag rhPEPD and rhPEPD were not remarkable. The level of p-Akt (Ser473) rose in prolidase-treated HaCaT cells in comparison with the control non-treated cells and the effects were comparable between His-Tag rhPEPD and rhPEPD treatment (Figure 7B). Similarly, the phosphorylation of ERK1/2 (Thr202/Tyr204) (Figure 7C) and STAT3 (Tyr705) (Figure 7D) was strong when prolidase was present in the cell culture medium, while in the non-treated cells it was weak. The biological activity of the recombinant human prolidase was also verified and confirmed at the study conducted in a model of inflammation in human keratinocytes [26].

## 4. Conclusions

In this paper, we present an optimized bacterial expression system and an optimized purification procedure for human recombinant prolidase. The expression system was based on the pET-16b plasmid which allowed the protein to be expressed fused to a histidine tag located at the N-terminus. The two-step rhPEPD purification procedure was based on (i) affinity chromatography on an Ni^2+^ ion-bound chromatography column (IMAC) and (ii) gel filtration with the possibility of tag removal by selective digestion with protease Xa. As a result, after the second purification step, high-purity prolidasewas obtained.

Our research showed that a high concentration of IPTG and high temperature of induction lead to a fast induction of gene expression, which as a result forces the host (bacterial cell) into an intensive and fast production of rhPEPD. The bacterial cell most likely perceives this process as unnatural and posing a threat. As a result of foreign gene expression, the produced protein may be recognized by the cell as toxic. Then, a defense mechanism is activated and the protein gets locked in an inclusion body. Obtained results show that slowing down the induction of gene expression by lowering the inducing factor, lowering the expression temperature, and extending the induction time leads to efficient protein production in the soluble fraction.

Moreover, the study proved that the presence of His-Tag changed neither the expression pattern of EGFR-downstream signaling proteins nor the prolidase catalytic activity.

## Figures and Tables

**Table 1:** List of primary antibodies used for Western immunoblotting.

Primary Antibodies	Dilution	Vendor
p44/42 MAPK (ERK1/2) Rabbit mAb	1:1000	Cell Signaling Technology
EGF Receptor Rabbit mAb	1:1000	Cell Signaling Technology
Phospho-EGF Receptor (Tyr1068) Rabbit mAb	1:1000	Cell Signaling Technology
Phospho-p44/42 MAPK (ERK1/2) (Thr202/Tyr204) Rabbit mAb	1:1000	Cell Signaling Technology
Akt Rabbit mAb	1:2000	Cell Signaling Technology
Phospho-Akt (Ser473) Rabbit mAb	1:1000	Cell Signaling Technology
STAT3Rabbit mAb	1:1000	Cell Signaling Technology
Phospho-STAT3 (Tyr705) Rabbit mAb	1:1000	Cell Signaling Technology
GAPDH Rabbit mAb	1:1000	Cell Signaling Technology

**Table 2:** Expression and purification conditions were tested.

Attempt	IPTG Final Concentration (mM)	Temperature (°C)	Time (h)	Protein Production	Lysis Buffers Additional Components	The Yield of Protein Production Insoluble Fraction
1	1	37	2	protein in inclusion bodies (in the pellet after centrifugation)	300 mM NaCl 20 mM Tris-HCl pH 8.0 20 mMimidazole 1 mM EDTA 10% glycerol 100 μg·mL^−^^1^ lysozyme 1–1.5% N-laurylsarcosine 2% Triton X-100	low
2		30	5			low
3		25	10			low
4		18	15			low
5		16	15			low
6	0.2	30	5	protein in the soluble fraction	300 mM NaCl 20 mM Tris-HCl pH 8.0 20 mMimidazole 1 mM EDTA 10% glycerol 100 μg·mL^−^^1^ lysozyme 1 mM TCEP	low
7		25	10			low
8		18	10			low
9		18	18			high
10		18	15			high

## Data Availability

Data supporting reported results are available upon request.
